# Reduced levels of reactive oxygen species correlate with inhibition of apoptosis, rise in thioredoxin expression and increased bovine leukemia virus proviral loads

**DOI:** 10.1186/1742-4690-6-102

**Published:** 2009-11-10

**Authors:** Amel Baya Bouzar, Mathieu Boxus, Arnaud Florins, Carole François, Michal Reichert, Luc Willems

**Affiliations:** 1 Université de Liège (ULg), Gembloux Agro-Bio Tech, Molecular and Cellular Biology, Gembloux, Belgium; 2 National Veterinary Research Institute, Pulawy, Poland; 3 Interdisciplinary Cluster for Applied Genoproteomics (GIGA), University of Liège (ULg), Liège, Belgium

## Abstract

**Background:**

Bovine Leukemia virus (BLV) is a deltaretrovirus that induces lymphoproliferation and leukemia in ruminants. In *ex vivo *cultures of B lymphocytes isolated from BLV-infected sheep show that spontaneous apoptosis is reduced. Here, we investigated the involvement of reactive oxygen species (ROS) in this process.

**Results:**

We demonstrate that (i) the levels of ROS and a major product of oxidative stress (8-OHdG) are reduced, while the thioredoxin antioxidant protein is highly expressed in BLV-infected B lymphocytes, (ii) induction of ROS by valproate (VPA) is pro-apoptotic, (iii) inversely, the scavenging of ROS with N-acetylcysteine inhibits apoptosis, and finally (iv) the levels of ROS inversely correlate with the proviral loads.

**Conclusion:**

Together, these observations underline the importance of ROS in the mechanisms of inhibition of apoptosis linked to BLV infection.

## Background

Bovine Leukemia Virus (BLV) is an oncogenic deltaretrovirus closely related to the primate T-lymphotropic viruses types 1-5 (i.e. HTLV-1 to -4 and STLV-1,-2,-3 and-5) [1-3]. Although successfully eradicated in some regions such as Europe, BLV is distributed worldwide. Infection remains mostly clinically silent with infected animals being referred to as asymptomatic or non-leukemic (AL) [4]. Only one-third of infected cattle develop a persistent lymphocytosis (PL) characterized by a permanent and relatively stable increase in the number of peripheral blood B lymphocytes co-expressing CD5, high levels of surface immunoglobulin M (sIgM), and myeloid markers [5,6]. The natural host for BLV is cattle which develops a fatal leukemia or a lymphoma in fewer than 5% of infected animals and after a long latency period (4-8 years) [7]. BLV can be transmitted experimentally to sheep which develop B-cell neoplasia with a higher incidence and after shorter latency periods than cattle [8]. This model may be helpful for understanding pathogenesis induced by the related human T-lymphotropic virus type I (HTLV-1).

We previously reported that the disruption of lymphocyte homeostasis results from a disequilibrium between cell proliferation and death [9,10]. Using BrdU (bromodeoxyuridine) and CFSE (carboxyfluorescein diacetate succinimidyl ester) labeling techniques, we showed that the dynamics of lymphocyte recirculation is unaltered but that the cell turnover is accelerated. Although these kinetic experiments are useful to explain the accumulation of infected cells *in vivo*, further understanding of BLV-induced pathogenesis requires dissection of metabolic pathways *in vitro*.

In this context, we have demonstrated that conditioned supernatants from BLV-infected peripheral blood mononuclear cell (PBMC) cultures can prevent uninfected cells from undergoing apoptosis. This so-called "indirect" protection against apoptosis involves glutathione [11] which is expected to buffer reactive oxygen species (ROS). On the other hand, when PBMCs isolated from infected sheep are transiently cultivated *ex vivo*, B-lymphocyte apoptosis is decreased compared to matched uninfected controls. This latter mechanism was referred to as "direct" protection against apoptosis [12,13]. In this work, we hypothesized that ROS might be important mediators of this process.

ROS are indeed implicated in the regulation of several cellular processes depending on their intracellular levels. A beneficial effect of ROS occurs in living cells at low/moderate concentrations and is associated with important physiological functions, including activation and modulation of signal transduction pathways, defense against infectious agents, induction of mitogenic response and regulation of mitochondrial apoptotic process [14,15]. In contrast, excessive levels of ROS are toxic to the cells causing damage to macromolecules such as lipids, proteins and nucleic acids [16,17]. Thus, oxidative stress results from an imbalance between ROS production and anti-oxidant activity conferred by enzymes like thioredoxin, glutathione peroxidase or glutathione reductase [18]. Alteration of the anti-oxidant system has been implicated both in malignant phenotypes as well as in carcinogenesis (see [19] for a review). For example, cancer cells are characterized by a more reducing environment and overexpression of the anti-apoptotic Bcl-2 factor which enhances resistance against ROS-induced-apoptosis [18].

Here, we evaluated the involvement of ROS in *ex vivo *apoptosis associated with BLV infection.

## Results

### The ROS levels are reduced in B cells from BLV-infected sheep

**Figure 1:**
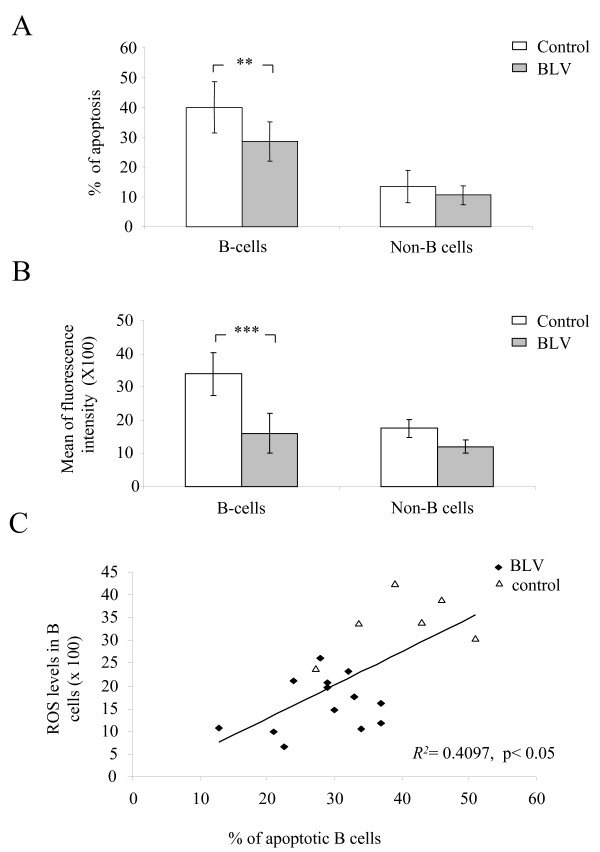
**Spontaneous apoptosis and ROS production in short term cultures**. A) Peripheral blood mononuclear cells (PBMCs) from BLV-infected (n = 13) and control (n = 6) sheep were isolated and cultivated for 24 h. B cells were labeled using anti-IgM monoclonal antibody (clone 1H4) and FITC-conjugated rabbit anti-mouse and fixed in ethanol. After staining with PI, hypodiploid cells (sub-G1 population) considered to be apoptotic were quantified by flow cytometry. Data are presented as the means of apoptotic rates ± standard deviation. ** denotes the statistical significance according to the non-paired Student's *t *test p < 0.01. B) PBMCs from BLV-infected (n = 13) and non-infected sheep (n = 6) were seeded in 24-well plates at a density of 10^6 ^cells/ml and incubated for 30 min at 37°C with 10 μM of CM-H_2_DCFDA. After 24 h of culture, B cells were stained using anti-IgM monoclonal (clone Pig45) and Alexa Fluor 647-conjugated donkey anti-mouse antibodies. The intracellular ROS levels were determined by flow cytometry and are presented as the mean fluorescence intensities (± standard deviation) of cellular chloromethyldichlorofluorescein (CM-DCF) within B and non-B lymphocyte populations. *** denotes the statistical significance according to the non paired Student's *t *test p < 0.001. C) Correlation between apoptotic rates and ROS levels measured in *ex vivo *cultures. Apoptotic rates and ROS levels were determined by flow cytometry as described in panels A and B, respectively. A correlation coefficient *R*^2 ^= 0.4097 was calculated from the linear regression analysis on percentages of apoptotic B cells and means of ROS fluorescence intensities. p < 0.05 denotes the statistical significance according to the non parametric Spearman test.

We and others previously reported that the percentages of B cells undergoing apoptosis in short term cultures are reduced in BLV-infected sheep [12,13]. We confirmed this observation in *ex vivo *cultures of peripheral blood mononuclear cells (PBMCs) isolated from a series of sheep (13 BLV-positive and 6 controls) whose hematological profiles are provided in Additional file 1. Fig. 1A indeed shows that the percentages of apoptotic B cells in PBMC cultures isolated from BLV-infected sheep are reduced compared to the controls (28.59% versus 39.96% respectively; p < 0.01). In contrast, the levels of spontaneous apoptosis were not significantly different (p = 0.19) in the non-B cell populations. These results thus confirm and extend previous reports in the literature [12,13]. Although the sub-G1 cells resulting from DNA fragmentation are the most specific hallmark of apoptosis, we confirmed these conclusions by Annexin V labeling experiments (data not shown).

We next measured the levels of intracellular reactive oxygen species (ROS) using the 2'-7'-dichlorofluorescein diacetate (CM-H_2_DCFDA) probe. The kinetics of ROS production after short term cultures (30 mn, 3 h and 6 h) were not significantly different in infected and control B cells (Additional file 2). After 24 h of culture, ROS-stimulated oxidation into 2'-7'-dichlorofluorescein was significantly lower (p < 0.001) in B cells isolated from BLV-infected PBMCs compared to controls (Fig. 1B). In contrast, ROS levels in non-B cells derived from BLV-infected and control cultures were not significantly different (p = 0.1, Fig. 1B). Importantly, reduction in B cell apoptosis correlates with decreased ROS production, the highest levels being observed in control cells (Fig. 1C, *R*^2 ^= 0.4097 and p < 0.05).

### Reduced ROS production in mitochondria of BLV-infected B lymphocytes

**Figure 2:**
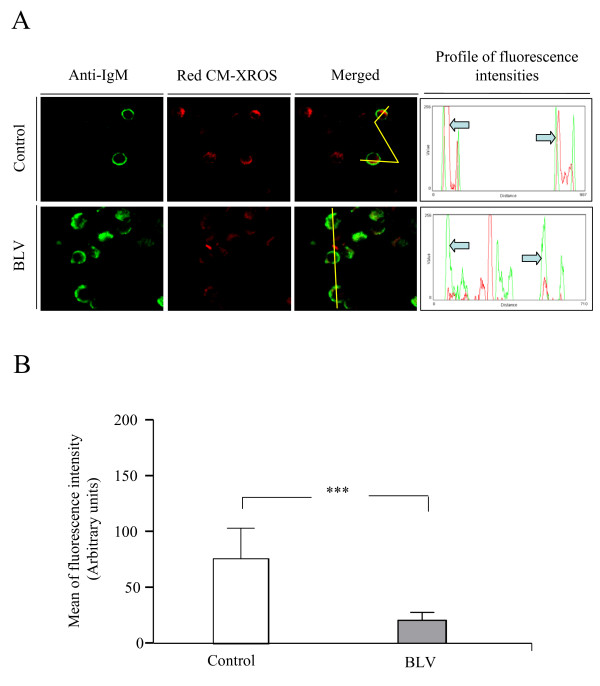
**Generation of ROS in B lymphocyte mitochondria**. Confocal microscopy analysis of ROS production in B cells isolated from 3 BLV-infected and 3 control sheep. 24 h after culture, PBMCs were labeled with 10 nM of Red CM-XROS probe and stained with anti-IgM monoclonal antibody (clone Pig45) and Alexa Fluor 488 goat anti-mouse conjugate. A) Confocal microscopy photographs and profiles of one representative experiment showing the fluorescence intensities of ROS (red fluorescence) and IgM (green fluorescence). For comparison, arrows indicate ROS levels (red) in B cells (green) in PBMCs from infected and control sheep. B) Quantification of arbitrary fluorescence intensities in 20 B cells from 3 BLV- infected and 3 control sheep. Data are presented as the means of fluorescence intensities (± standard deviation). *** denotes the statistical significance according to the non paired Student's *t *test p < 0.001.

Although the CM-H_2_DCFDA probe is routinely used to assess ROS production in all cell compartments, we confirmed our results using the fixable probe, MitoTracker Red CM-XROS, which specifically quantifies ROS levels directly within mitochondria. Upon oxidation, red fluorescence revealed by confocal microscopy was reduced in B lymphocytes isolated from BLV-infected sheep and cultivated for 24 h (compare arrows in fluorescence intensity profiles of one representative experiment shown in Fig. 2A). Quantification in 20 randomly selected B cells from 3 BLV- infected and 3 control sheep demonstrated that the means of fluorescence intensities in mitochondria of BLV-infected B lymphocytes were indeed significantly lower than in controls (Fig. 2B, p < 0.001).

These observations thus confirm that, upon cultivation, B cells isolated from BLV-infected sheep produce lower levels of ROS compared to uninfected controls.

### DNA oxidative damage is reduced in B lymphocytes of BLV-infected sheep

**Figure 3:**
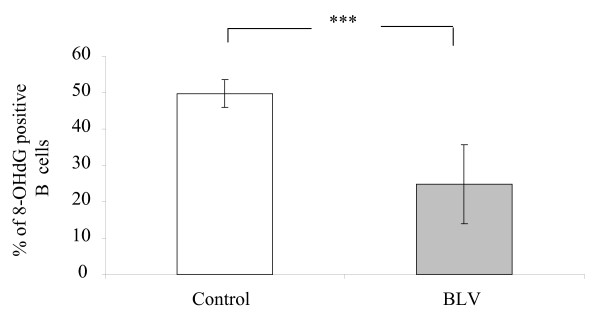
**Analysis of DNA oxidative damage**. After 24 h of culture of PBMCs from BLV-infected (n = 3) and control (n = 3) sheep, cells were stained for IgM and 8-hydroxy-2'-deoxyguanosine (8-OHdG) and analyzed by flow cytometry. Data are presented as the percentages of 8-OHdG positive B cells (± standard deviation) in the total B cell population. *** denotes the statistical significance according to the non paired Student's *t *test p < 0.001.

ROS species may cause a series of alterations to cell components such as DNA, proteins or lipids. Oxidative DNA damage can be measured by using the 8-hydroxy-2'-deoxyguanosine (8-OHdG) product [20]. Therefore, we determined the percentages of 8-OHdG positive B cells in PBMC cultures isolated from 3 BLV-infected and 3 control sheep (Fig. 3). Flow cytometry analysis revealed that B-lymphocytes staining for 8-OHdG were significantly lower in BLV-infected cultures compared to controls (Fig. 3, p < 0.001). These results demonstrate that a major product of oxidative DNA (8-OHdG) is reduced in B cells isolated from BLV-infected sheep, providing a biological relevance to ROS activity.

### BLV-infected B cells express high levels of thioredoxin

**Figure 4:**
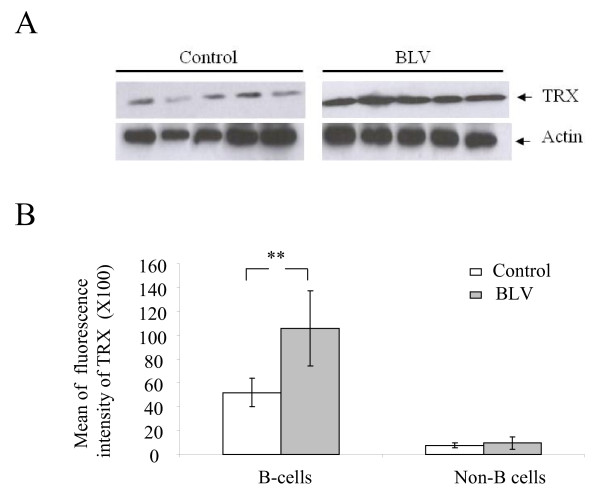
**Analysis of thioredoxin expression**. A) Western blot analysis of thioredoxin (TRX) expression in PBMCs isolated from BLV-infected (n = 5) and control (n = 5) sheep using a goat polyclonal antibody specific for TRX. Actin was analyzed in parallel as a loading control. B) Immunofluorescence staining of thioredoxin was performed with 10^6 ^PBMCs/ml isolated from BLV-infected (n = 8) and control (n = 5) sheep. After B cell staining using anti-IgM monoclonal antibody (clone Pig45) and Alexa Fluor 647-conjugated donkey anti-mouse antibodies, cells were fixed in paraformaldehyde (4%) and permeabilised with PBS/TritonX-100 (0.5%). Cells were then incubated with anti-human TRX monoclonal antibody and Alexa Fluor 488 goat anti-mouse conjugate and analyzed by flow cytometry. Results are presented as the means of fluorescence intensities (± standard deviation) of TRX in B and non-B cells. ** denotes the statistical significance according to the non paired Student's *t *test p < 0.01.

We next analyzed the levels of the thioredoxin (TRX) antioxidant protein. Western blot analysis of Fig. 4A show that, compared to controls, thioredoxin was overexpressed in PBMCs isolated from BLV-infected sheep. Consistently, thioredoxin fluorescence intensities measured by flow cytometry were significantly higher in BLV-infected B cells (Fig. 4B, p < 0.01). In contrast, thioredoxin levels were not significantly different in non-B cell populations (Fig. 4B, p = 0.47).

These results demonstrate that B cells isolated from BLV-infected sheep express high levels of thioredoxin.

### Valproate (VPA)-induced apoptosis of BLV-infected B lymphocytes involves a ROS-dependent pathway

**Figure 5:**
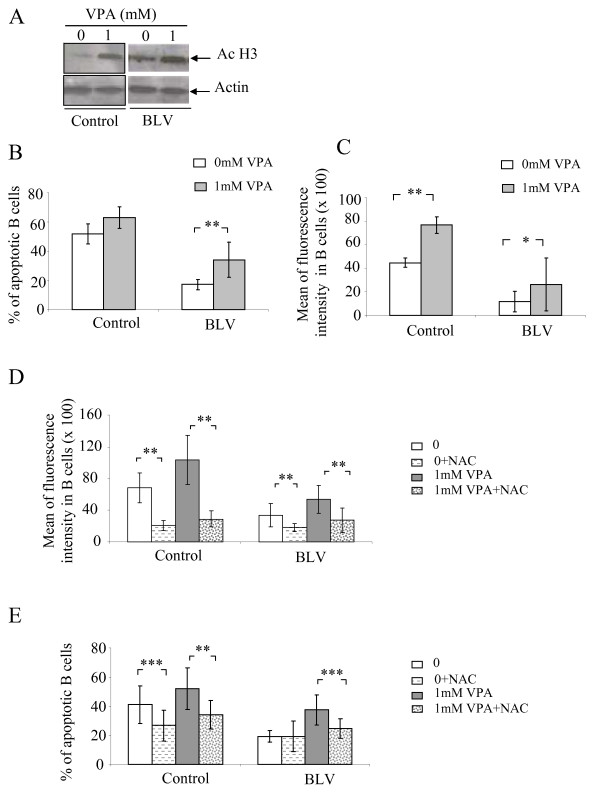
**Effect of VPA on apoptosis and ROS production**. PBMCs from BLV-infected and control sheep were isolated and cultivated for 24 h in the absence (0) or the presence of 1 mM of VPA. A) Representative Western blot analysis using an antibody specific for the acetylated form of histone H3. Actin was analyzed in parallel as a loading control. B) The extent of B cell apoptosis was measured by determining the level of nuclear DNA fragmentation. B lymphocytes from 6 BLV-infected and control sheep were labeled using an anti-IgM monoclonal antibody (clone 1H4) and a FITC-conjugated rabbit anti-mouse antiserum (Becton Dickinson). After ethanol fixation and propidium iodide staining, hypodiploid B lymphocytes (i.e. in sub-G1) considered to be apoptotic were quantified by flow cytometry. ** denotes the statistical significance of the differences between 0 and 1 mM VPA, accordingly to the paired Student's *t *test p < 0.01. C) ROS levels were evaluated in parallel after incubation of PBMCs with 10 μM of CM-H_2_DCFDA probe prior to VPA treatment. Data represent the mean fluorescence intensities (± standard deviation) of cellular chloromethyldichlorofluorescein (CM-DCF). * and ** denote the statistical significance of the differences between 0 and 1 mM VPA, accordingly to the paired Student's *t *test (p < 0.05 and p < 0.01, respectively). D and E) PBMCs from BLV-infected and control sheep were cultivated for 24 h in absence or presence of the free radical scavenger N-acetyl-L-cysteine (NAC) added 2 h prior VPA treatment (1 mM). Cells isolated from BLV-infected and control sheep were analyzed by flow cytometry to determine the levels of intracellular ROS (D) and the rates of apoptosis (E). ** and *** denote the statistical significances according to the paired Student's *t *test (p < 0.01 and p < 0.001, respectively).

We previously reported an approach to treat leukemia in BLV-infected sheep using the VPA histone deacetylase inhibitor [21]. Since we showed that VPA acts at least in part through induction of apoptosis, we investigated the involvement of ROS in VPA therapy. As control, we first analyzed histone H3 acetylation in presence of a therapeutic dose of 1 mM VPA as previously described [22]. As expected for an HDAC inhibitor, VPA induced the hyperacetylation of histone H3 (Ac H3) (Fig. 5A). At the concentration of 1 mM, VPA was also proapoptotic for B lymphocytes isolated from controls and BLV-infected sheep (Fig. 5B), confirming our previous results [21]. To determine the effect of VPA on ROS production, PBMCs from BLV-infected (n = 6) and control sheep (n = 6) were preincubated for 30 min at 37°C with 10 μM of CM-H_2_DCFDA and then cultivated for 24 h with 1 mM of VPA. Cell cultures undergoing increased apoptosis upon VPA treatment also yielded increased ROS production (Fig. 5C). We concluded that VPA-induced apoptosis parallels ROS production.

To correlate VPA-induced apoptosis in B lymphocytes and reactive oxygen species, ROS production was inhibited with 10 mM of the free radical scavenger *N*-acetyl cysteine (NAC). As expected, NAC treatment resulted in a significant inhibition of ROS in the presence of VPA in B cells isolated from BLV-infected (Fig. 5D, p < 0.01) and control sheep (Fig. 5D, p < 0.01). Concomitantly, NAC also efficiently inhibited apoptosis of B lymphocytes isolated from BLV-infected (Fig. 5E, p < 0.001) and control sheep (Fig. 5E, p < 0.01). Collectively, these results show that VPA-induced apoptosis in BLV-infected and control B lymphocytes involves a ROS dependent pathway.

### ROS levels correlate inversely with proviral load

**Figure 6:**
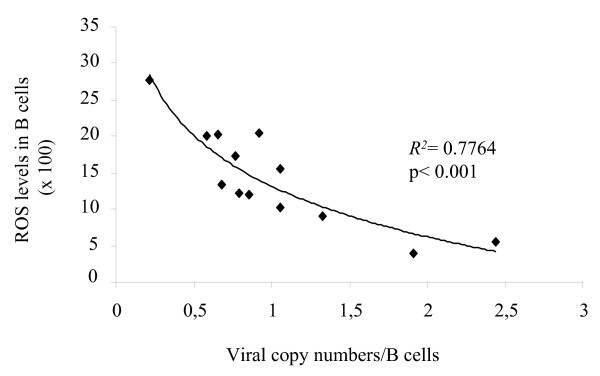
**Correlation between ROS levels and proviral loads**. Proviral loads (expressed as numbers of viral copies in B cells) were measured by real-time PCR in PBMCs isolated from BLV-infected sheep (n = 13). ROS levels were measured in B cells cultivated for 24 h using the CM-H_2_DCFDA probe. A correlation coefficient *R*^2 ^= 0.7764 was calculated. p < 0.001 was determined using the non parametric Spearman test.

Finally, we measured the proviral loads in PBMCs isolated from 13 BLV-infected sheep using real-time PCR as described previously [23]. In parallel, the intracellular ROS levels were monitored upon cultivation of the same samples using the CM-H_2_DCFDA probe. As shown in Fig. 6, ROS levels in B lymphocytes inversely correlated with the proviral loads (correlation coefficient *R*^2 ^= 0.7764 and p < 0.001). These results show that spontaneous ROS production is reduced in B cells from animals with high proviral loads.

## Discussion

We and others previously reported that extent of apoptosis is reduced in *ex vivo *cultures [12,13], suggesting that the lifespan of BLV-infected B cells may also be prolonged *in vivo*. However, the mechanisms involved are insufficiently understood. In this study, we provide new evidence demonstrating that ROS play a key role in inhibition of apoptosis associated with BLV infection. Decreased levels of ROS as evidenced by two different fluorescent probes were detected in B cells isolated from BLV-infected sheep. A direct marker of ROS-induced DNA damage, 8-OHdG was also reduced, while thioredoxin was overexpressed, further validating the antioxidant status observed in BLV-infected B cells. We also showed that VPA treatment increases ROS levels and concomitantly induces apoptosis. Inversely, the scavenging of ROS with NAC decreased apoptotic rates. Finally, spontaneous ROS production inversely correlates with proviral loads measured *in vivo*.

Very little is known about the involvement of the ROS pathway in BLV infection. To our knowledge, a single report showed that the addition of low concentrations of H_2_O_2 _(10-20 μM) increased BLV capsid protein (p24) expression in BLV-infected cell lines [24]. Higher concentrations of H_2_O_2 _(200-300 μM) inhibited proliferation as well as p24 expression, and induced apoptosis. Our present study is the first that correlates spontaneous *ex vivo *apoptosis, ROS production and proviral loads in BLV-infected sheep.

As a preliminary remark, we should first emphasize that in the absence of culture or after short culture intervals (<6 h), similar ROS levels are measured in BLV-infected and control B cells (Additional file 2). Although it is possible that viral replication and/or expression are prerequisites for ROS inhibition *ex vivo*, a more likely interpretation is that survival *in vivo *of both uninfected and BLV-infected B lymphocytes requires adequate ROS levels to persist in the sheep.

A second important point to address concerns the apparent conflict between conclusions drawn from *ex vivo *and *in vivo *observations. Why should BLV-infected cells (thus less prone to undergo *ex vivo *apoptosis) have a shorter lifespan *in *vivo [9,10]? The most straightforward interpretation is that BLV-infected lymphocytes are indeed more resistant to apoptosis *in vivo *but that these cells are efficiently destroyed by the host immune response. According to this model, there is thus no contradiction between inhibition of apoptosis *ex vivo *and increased cell turnover *in vivo*. This interpretation is also compatible with the fact that ROS levels correlate positively with apoptosis (Fig. 1C) and negatively with proviral loads (Fig. 6).

The balance between beneficial and deleterious effects of ROS is believed to be critical for cell survival and is achieved by a mechanism called "redox homeostasis" [25]. As a defense against oxidative stress, cells possess several antioxidant enzymes such as superoxide dismutase, catalase, glutathione peroxidase and thioredoxin (TRX) [18]. Altered expression of these proteins and disruption of ROS homeostasis has been involved in the development of virus-induced malignancies [26]. Intracellular overexpression of TRX was detected in cells transformed by oncoviruses including HTLV-1, Epstein-Barr and Human Papilloma virus [27-29]. HTLV-1 transformed T lymphocytes constitutively release high levels of TRX that increase upon addition of H_2_O_2;_; this limits ROS production and protects cells from apoptosis [30]. On the other hand, deregulation of the TRX system associated with low levels of glutathione has been largely implicated in the physiopathology of HIV-1 [31-33]. In fact, the anti-apoptotic function of glutathione inversely correlates with the terminal lymphodepletion observed during the AIDS phase. HTLV-1-infected cell survival depends on the balanced effects of Tax, p13 and p12 regulatory proteins (reviewed in [34]). Two of these proteins, Tax and p13, have opposite effects on the redox state regulation. Indeed, p13 increases ROS production via mitochondria depolarization resulting in an increase of cell death [35]. Inversely, HTLV-1 Tax stimulates thioredoxin expression which results in ROS decrease and subsequently growth of transformed cells [36,37]. Our data demonstrate that B cells isolated from BLV-infected sheep overexpress thioredoxin (Fig. 4B). However, our preliminary results indicate that thioredoxin expression does not appear to be directly controlled by BLV Tax protein (data not shown).

The development of strategies to modulate cell signaling by restoring "redox homeostasis" may be a promising anti-cancer therapy. Indeed, some compounds such as the SAHA histone deacetylase inhibitor can target the thioredoxin system leading to ROS generation and induction of apoptosis in cancer cells (for review, see [38]). HDAC inhibitors are known to be proapoptotic for cells infected by HTLV-1 [39], EBV [40] and HPV [41]. In a previous report [21], we demonstrated that VPA induces apoptosis of BLV-infected B cells and hypothesized that this effect was mediated at least in part through HDAC inhibition. We now show that VPA increases ROS production in BLV-infected B cells providing a non exclusive alternative mode of action. However, VPA treatment of PBMCs isolated from BLV-infected and control sheep had no effect on thioredoxin expression (data not shown), suggesting that VPA acts through another antioxidant pathway. Importantly, the free radical scavenger NAC concomitantly abrogates ROS production and VPA-induced apoptosis indicating that the intracellular oxidative state directly triggers sensitivity to VPA. In fact, HDAC inhibition and ROS stimulation may be linked through chromatin relaxation and transcriptional activation of specific cellular genes. In particular, VPA has been shown to activate redox-sensitive transcription factors (Nrf2) and their subsequent interaction with the anti-oxidant response element (ARE) on specific gene promoters [42].

In conclusion, we have demonstrated in this report that direct inhibition of apoptosis by bovine leukemia virus correlates with reduced production of reactive oxygen species *ex vivo*, a mechanism that implicates the thioredoxin system. Our results thus provide evidence linking inhibition of apoptosis and reduced ROS levels resulting in a low DNA oxidative damage in BLV-infected B cells. These data are consistent with a virus-associated ROS scavenging mechanism conferring a reducing environment and interfering with apoptosis of the infected cell. Finally, it is noteworthy that resistance to oxidative stress *ex vivo *correlates with increased proviral loads *in vivo*.

## Methods

### Experimental animals

A total of 13 BLV-infected sheep and 7 controls used in this study were maintained under restricted conditions at the Agricultural University of Gembloux (Belgium) and the National Veterinary Research Institute of Pulawy (Poland). Sheep # 4, 5, 6, 12, 13, 15, 16, 17, 18, 19, 4219, 5183 and 5193 were infected by a wild type proviral clone of BLV strain 344 (pBLV344) [43]. All infected animals were either in the asymptomatic stage of the disease (total leukocyte counts ranging below 10,000 cells per mm^3^) or lymphocytic (above 10,000 cells per mm^3^). Sheep # 5050, 5209, 7053, 7057, 9, B20 and M21 were used as uninfected controls.

### Isolation of PBMCs and *ex vivo *culture

Peripheral blood mononuclear cells (PBMCs) were isolated by density-gradient centrifugation using Percoll (*GE Healthcare*). Briefly, venous blood was collected by jugular venipuncture and mixed with EDTA used as an anticoagulant (1 ml of 7.5% EDTA per 25 ml of blood). PBMCs were washed twice with phosphate-buffered saline (PBS) supplemented with 0.075% EDTA and three time with PBS alone to deplete platelets. After estimation of cell viability by trypan blue dye exclusion, 10^6 ^cells were seeded in 24 well plates and cultivated for 24 h at 37°C in a humidified, 5% CO_2_-air incubator in RPMI 1640 medium (*Lonza*) supplemented with 10% of fetal calf serum (FCS), 2 mM L-glutamine, 100 U of penicillin and 100 μg of streptomycin (*Lonza*). PBMCs isolated from non infected sheep were used as controls. To determine the percentage of B lymphocytes, PBMCs were labeled with anti-IgM monoclonal antibody (clones 1H4 or Pig45) and with FITC-conjugated rabbit anti-mouse (*Dako*) or Alexa Fluor 647-conjugated donkey anti-mouse antibodies (Invitrogen), respectively. Cells were then analyzed by flow cytometry using a FacsAria apparatus (*Becton Dickinson*).

### Detection of apoptosis

The B cell apoptotic rates were determined by evaluating DNA fragmentation using propidium iodide (PI) staining. Briefly, B cells were labeled using anti-IgM monoclonal (clone 1H4) and FITC-conjugated rabbit anti-mouse (*Becton Dickinson*) antibodies, and fixed in 70% ethanol at -20°C for at least 1 h. After two washes, cells were treated with RNAse A (50 μg/ml) (*Sigma/Aldrich*) for 30 min at 37°C, centrifuged and resuspended in PBS containing 20 μg/ml propidium iodide (*Sigma/Aldrich*). The percentage of B-cells undergoing nuclear DNA fragmentation was then quantified using a FacsAria flow cytometer (*Becton Dickinson*). Cell aggregates were excluded from the analysis using the FSC-A/FSC-H gating method. Ten thousand events were collected and analyzed with the FacsDiva software. Data are given in percentages of hypodiploid B-cells staining at sub-G1 fluorescence intensity and reflecting the number of B-apoptotic cells. To determine the effect of VPA on apoptosis, PBMCs (1.10^6^) isolated from BLV-infected and control sheep were cultivated in the absence and the presence of 1 mM VPA (*Sigma/Aldrich*) and the percentages of apoptotic B cells were determined as described above.

### Analysis of reactive oxygen species (ROS) production

Intracellular ROS levels were monitored using 5,6-chloromethyl-2',7'-dichlorodihydrofluorescein diacetate acetyl ester (CM-H_2_DCFDA, *Invitrogen*), a probe which is oxidized to 2'-7'-dichlorofluorescein upon ROS stimulation. PBMCs from BLV-infected and control sheep were seeded in 24-wells plates at a density of 10^6^cells/ml and incubated for 30 min at 37°C with 10 μM of CM-H_2_DCFDA. After 24 h of culture, B cells were stained using anti-IgM monoclonal (clone Pig45) and Alexa Fluor 647-conjugated donkey anti-mouse antibodies (*Invitrogen*). Cells were then collected, washed with PBS and analyzed with a FacsAria flow cytometer. The fluorescence intensity of chloromethyldichlorofluorescein (CM-DCF) was detected at a wavelength of 530 nm after a laser excitation beam of 480 nm. B cell specific staining was measured at a wavelength of 650 nm after excitation with a 630 nm laser beam. To determine the effect of VPA on ROS production, PBMCs from BLV-infected and control sheep were preincubated for 30 min at 37°C with 10 μM CM-H_2_DCFDA and then cultivated with VPA (1 mM). For ROS inhibition experiments, cells were treated with 10 mM of the free radical scavenger N-acetyl-L-cysteine (NAC) (*Calbiochem*) 2 h prior to incubation with the CM-H_2_DCFDA probe.

### Measurement of ROS production in B cell mitochodria

Production of ROS in mitochondria was measured using MitoTracker Red CM-XROS (*Molecular probes*), a derivative of X-rosamines that accumulates in mitochondria and produces a red fluorescent signal upon oxidation with H_2_O_2_. PBMCs from BLV-infected and control sheep were cultivated in 24-well plates containing poly lysine-coated 12-mm glass coverslips. 24 h later, cells were incubated for 45 min with 10 nM of Red CM-XROS probe, washed and fixed in paraformaldehyde/PBS 4/100 (v/v). B cells were stained using anti-IgM monoclonal (clone Pig45) and Alexa Fluor 488-conjugated goat anti-mouse antibodies (Invitrogen). After mounting coverslips in Tris Buffered Saline (TBS)/Glycerol, cells were analyzed by confocal fluorescence microscopy (Axiovert 200 microscope with a LSM 510 confocal device; *Carl Zeiss Microscope*) using argon (488 nm) and HeNe (633 nm) lasers.

### Measurement of DNA oxidative damage in B cells

The extent of DNA damage resulting from oxidative stress was evaluated by quantification of the major oxidative DNA product: 8-hydroxy-2'-deoxyguanosine (8-OHdG), using the OxyDNA assay Kit (*Calbiochem*) according to the manufacturer's instructions. Briefly, PBMCs from BLV-infected and control sheep were cultivated for 24 h at a density of 10^6 ^cells/ml. After staining of B cells using anti-IgM monoclonal (clone Pig45) and Alexa Fluor 647-conjugated donkey anti-mouse antibodies (*Invitrogen*), cells were fixed in paraformaldehyde/PBS 4/100 (v/v) and permeabilised with PBS/TritonX-100 5/1000 (v/v). After a final wash, cells were labeled for 1 h with an 8-oxoguanine specific FITC-conjugate and analyzed by flow cytometry using a FacsAria apparatus.

### Flow cytometry analysis of thioredoxin

After B cell staining using anti-IgM monoclonal (clone Pig45) and Alexa Fluor 647-conjugated donkey anti-mouse antibodies (*Invitrogen*), cells were fixed in paraformaldehyde/PBS 4/100 (v/v), permeabilised with PBS/TritonX-100 5/1000 (v/v) and labeled with an anti-human TRX monoclonal antibody (*BD Pharmingen*) and with Alexa Fluor 488-conjugated goat anti-mouse antibodies (*Invitrogen*). Mean fluorescence intensities of thioredoxin levels were measured by flow cytometry using a FacsAria apparatus.

### Western immunoblotting of acetylated histone H3 and thioredoxin expression

PBMCs from BLV-infected and control sheep were cultivated for 24 h in the absence or the presence of 1 mM of VPA. Cells (5 × 10^6^) were harvested by centrifugation at 500 g for 10 min at 4°C and washed with ice-cold PBS, then lysed by incubation for 30 min on ice in RIPA buffer (50 mM Tris pH 7.5, 150 mM NaCl, 0.1% SDS, 1% NP-40, 0.5% sodium deoxycholate, supplemented with *Complete *protease inhibitors, *Roche*). After centrifugation at 13,000 *g *for 10 min, the supernatant was stored at -80°C. Proteins were quantified using Micro-BCA protein assay kit (*Pierce*) and 20 μg were electrophoresed on a 15% SDS-polyacrylamide gel and blotted onto a nitrocellulose membrane (*GE Health*). Filters were incubated overnight at 4°C in presence of anti-acetylated histone H3 (*Upstate Cell Signaling*), anti-TRX (*Santa Cruz Biotechnology*) or anti-actin (*Sigma/Aldrich*) antibodies followed by subsequent treatment with horseradish peroxidase conjugates (*Santa Cruz Biotechnology*) for 1 h at room temperature. Immunoreactive bands were visualized using the *ECL Advance *western blot detection reagent (*GE Health*).

### Determination of proviral load

Genomic DNA was extracted from PBMCs of BLV-infected sheep using the DNeasy Kit (*Quiagen*) according to the manufacturer's instructions. One hundred nanograms of genomic DNA were used for real-time PCR amplification of BLV proviral sequences. A segment corresponding to the *pol *gene (nucleotide 3994 to 4060) was amplified with a final concentration of 900 nM of 2 primers: (5'-GAAACTCCAGAGCAATGGCATAA-3' and 5'-GGTTCGGCCATCGAGACA-3') and revealed with a Minor Groove Binder (MGB) fluorescent probe (250 nM of 6-carboxyfluorescein-CTCACCCACTGCAAC-MGB) using the TaqMan PCR universal master mix with a StepOne apparatus (*Applied Biosystems*). A standard curve was generated after amplification of defined copy numbers (from 1 to 10^7 ^of plasmid pBLV344) with 100 ng of control genomic DNA. To correct for differences in DNA concentrations and amplification efficiencies between samples, the 18S ribosomal DNA was amplified in parallel using a final concentration of 900 nM of the following pair of primers: (5-TTGGATAACTGTGGTAATTCTAGAAGCTAA-3' and 5'-CGGGTTGGTTTTGATCTGATAAAT-3') and 250 nM of the 6-carboxyfluorescein-CATGCCGACGGGCGCTGA-MGB probe. The proviral loads were finally normalized to the percentage of B lymphocytes.

### Statistical analysis

Statistical significances of experimental data were analyzed using the Student's *t *test and the non parametric Spearman test. The observed differences between experimental results were stated as statistically significant, highly statistically significant and very highly statistically significant when p < 0.05 (*), p < 0.01 (**) and p < 0.001 (***), respectively.

## Competing interests

The authors declare that they have no competing interests.

## Authors' contributions

ABB designed the experiments, performed most of the analyzes, and drafted the paper. MB participated in the confocal microscopy analyzes. CF provided technical help. AF and MR performed sheep experimentation. LW designed the experiments, supervised studies, and edited the manuscript.

## Supplementary Material

Additional file 1 Sheep hematological profiles. Click here for file

Additional file 2 **Kinetics of ROS production in B cells**. PBMCs isolated from BLV-infected (n = 13) and non-infected (n = 7) sheep were seeded in 24-well plates at a density of 10^6 ^cells/ml and incubated for 30 min at 37°C with 10 μM of CM-H_2_DCFDA. After 3 h and 6 h of culture, B cells were stained using anti-IgM monoclonal (clone Pig45) and Alexa Fluor 647-conjugated donkey anti-mouse antibodies. The intracellular ROS levels were determined by flow cytometry and are presented as the mean fluorescence intensities (± standard deviation) of cellular chloromethyldichlorofluorescein (CM-DCF) within B cell populations. Click here for file
