# Supplementary material for: Reduced levels of reactive oxygen species correlate with inhibition of apoptosis, rise in thioredoxin expression and increased bovine leukemia virus proviral loads
Source: Retrovirology. 2009 Nov 10;6:102. doi: 10.1186/1742-4690-6-102 (PMC2779800; doi:10.1186/1742-4690-6-102)
Supplement: Additional file 1 — Sheep hematological profiles. [file 1742-4690-6-102-S1.doc]

**Additional file 1 : Sheep hematological profiles**

| #Sheep | leucocytes  cells/mm³ | Lymphocytes % | B cells % | Monocytes % | Granulocytes % | Red blood cells/mm³ | Proviral load copy number/ B cells |
| --- | --- | --- | --- | --- | --- | --- | --- |
|
|
|
| **Infected sheep** | | | | | | | |
| 4 | 11900 | 64 | 28.7 | 7 | 29 | 9220 | 1.05 |
| 5 | 10600 | 62 | 44.4 | 8 | 30 | 8980 | 0.68 |
| 6 | 9900 | 68 | 44.5 | 7 | 25 | 8130 | 0.85 |
| 12 | 6700 | 54 | 25 | 6 | 38 | 7760 | 0.65 |
| 13 | 16800 | 60 | 49.6 | 10 | 30 | 8270 | 1.05 |
| 15 | 2200 | 69 | 30.8 | 4 | 27 | 5490 | 0.76 |
| 16 | 3800 | 77 | 52.4 | 5 | 18 | 5350 | 0.58 |
| 17 | 8400 | 64 | 39.3 | 8 | 28 | 9660 | 1.91 |
| 18 | 12500 | 60 | 48.7 | 8 | 32 | 9440 | 2.44 |
| 19 | 1700 | 60 | 57.5 | 5 | 35 | 5350 | 1.32 |
| 5183 | 91002 | 39.9 | 57.2 | 19.5 | 41.2 | 4130 | 0.79 |
| 5193 | 17200 | 77.4 | 45 | 8.2 | 14.4 | 11680 | 0.92 |
| 4219 | 9300 | 59.4 | 16.5 | 4.1 | 36.5 | 10900 | 0.21 |
| **Non infected sheep** | | | | | | | |
| 5209 | 4460 | 75 | 20.8 | 7.5 | 17.5 | 12210 | 0 |
| 5050 | 3130 | 50.4 | 27.1 | 3.6 | 46 | 8010 | 0 |
| 7053 | 6500 | 73.5 | 17 | 2.9 | 23.6 | 14400 | 0 |
| 7057 | 3770 | 66.8 | 16.7 | 4.9 | 28.3 | 8970 | 0 |
| 9 | 6200 | 54 | 16.7 | 6 | 40 | 9650 | 0 |
| B20 | 4100 | 12.8 | 12.8 | 9 | 31 | 7450 | 0 |
| M21 | 4200 | 18.6 | 18.6 | 9 | 33 | 9003 | 0 |
