# Supplementary material for: Reduced levels of reactive oxygen species correlate with inhibition of apoptosis, rise in thioredoxin expression and increased bovine leukemia virus proviral loads
Source: Retrovirology. 2009 Nov 10;6:102. doi: 10.1186/1742-4690-6-102 (PMC2779800; doi:10.1186/1742-4690-6-102)
Supplement: Additional file 2 — Kinetics of ROS production in B cells. PBMCs isolated from BLV-infected (n = 13) and non-infected (n = 7) sheep were seeded in 24-well plates at a density of 106 cells/ml and incubated for 30 min at 37°C with 10 μM of CM-H2DCFDA. After 3 h and 6 h of culture, B cells were stained using anti-IgM monoclonal (clone Pig45) and Alexa Fluor 647-conjugated donkey anti-mouse antibodies. The intracellular ROS levels were determined by flow cytometry and are presented as the mean fluorescence intensities (± standard deviation) of cellular chloromethyldichlorofluorescein (CM-DCF) within B cell populations. [file 1742-4690-6-102-S2.doc]

**Additional file 2 : Kinetics of ROS production in B cells**

Kinetics of ROS production in B cells isolated from BLV-infected (n=13) and non-infected (n=7) sheep peripheral blood mononuclear cells (PBMCs). PBMCs were seeded in 24-well plates at a density of 10^6^ cells/ml and incubated for 30 min at 37°C with 10μM of CM-H_2_DCFDA. After 3h and 6h of culture, B cells were stained using anti-IgM monoclonal (clone Pig45) and Alexa Fluor 647-conjugated donkey anti-mouse antibodies. The intracellular ROS levels were determined by flow cytometry and are presented as the mean fluorescence intensities (± standard deviation) of cellular chloromethyldichlorofluorescein (CM-DCF) within B cell populations.
